# Hierarchical object combination and tool use in the great apes and human children

**DOI:** 10.1007/s10329-022-01003-2

**Published:** 2022-08-01

**Authors:** Misato Hayashi, Hideko Takeshita

**Affiliations:** 1 Chubu Gakuin University, 30-1 Naka Oida-cho, Kakamigahara, Gifu 504-0837 Japan; 2 Japan Monkey Centre, Inuyama, Aichi Japan; 3 Otemon Gakuin University, Ibaraki, Osaka Japan

**Keywords:** Object manipulation, Material intelligence, Great apes, Humans, Hierarchical complexity, Cognitive development

## Abstract

Object manipulation can be used as a comparative scale of cognitive development among primates, including humans. Combinatory object manipulation is a precursor of tool-using behavior that indicates material intelligence in primates. However, developmental data on it regarding the great apes other than chimpanzees is insufficient. We conducted a longitudinal investigation of humans and chimpanzees as well as a cross-sectional examination of other great-ape infants (two bonobos, three gorillas, and four orangutans) in captive settings by using two kinds of tasks that required either inserting or stacking combinatory action. The four species of great apes and humans demonstrated both types of combinatory object manipulation during infancy. However, the order of development in different types of combinatory object manipulations varied among the great apes. Furthermore, we applied a nesting-cup task to examine the hierarchical complexity in the combinatory strategies of human children and adult chimpanzees. Both of them exhibited highly hierarchical combinations in the nesting-cup task and employed the subassembly strategy, indicating that an action merge may exist not only in human children but also in adult chimpanzees. The results were discussed with reviews of the tool-use literature from the wild great apes. The early acquisition of an inserting action in the chimpanzees may explain the tool utilization commonality reported in wild chimpanzees. The combinatory object manipulation may have worked as an external enhancer to achieve an additional hierarchical complexity in cognition and behavior, eventually leading humans to develop a language system.

**Supplementary Information:**

The online version contains supplementary material available at 10.1007/s10329-022-01003-2.

## Introduction

What is the evolutionary underpinning of the human language? Comparing cognition in human and nonhuman primates of different developmental stages using a unitary scale would provide valuable clues to answer this question (Vauclair and Bard 1983; Hayashi and Matsuzawa 2003; Hayashi and Takeshita 2021). Fujita and Fujita (2021, in this volume) discussed the evolutionary continuity between human language and nonhuman animals’ cognitive capacities by applying a linguistic term, “Merge,” as a key concept. Merge has been illustrated as an important issue in human linguistics (Berwick et al. 2013) and can be defined as a recursive operation that takes two syntactic objects and forms an unordered set. If we apply this term to the manipulatory actions of primates, including humans, merge in human language corresponds to combinatory object manipulations, which is a precursor of tool-using behavior.

## Combinatory object manipulation

Combinatory object manipulation has been studied in cross-species comparison (Torigoe 1985) and from developmental perspectives (Takeshita 2001). Torigoe (1985) reported that among 74 species of tested primates, great apes exhibited the highest repertoire of combinatory object manipulation (identified as ‘secondary manipulation’ in the study), followed by cebus monkeys, macaques, guenons, mangabeys, and baboons, although the other species did not combine objects during manipulative actions. Takeshita (2001) reported that infant chimpanzees, aged 2–4 years, readily exhibited combinatory object manipulation comparable to that of 1-year-old human infants. Moreover, both infant chimpanzees and humans during the language-acquisition period exhibited similar motor characteristics: repetition, adjustment, and reversal of actions and shifts of attention. Based on the fundamental ability of combinatory object manipulation, some primates proceeded to use an object as a tool in their natural habitat. The rich repertoire of tool-using behaviors indicates high levels of cognitive development and material intelligence that characterizes the chimpanzee and human lineages (McGrew 2010, 2013). The first part of the present study aimed to add developmental data on object manipulation in four species of great apes and human children to investigate their cognitive development with special focus on two types of combinatory object manipulation: insertion and stacking. The second part examined the levels of hierarchical complexity inherent in object manipulation and the grammatical strategies applied in combining objects in humans and chimpanzees.

## Object-manipulation tasks as a comparative scale

Object manipulation has been employed as a comparative scale of cognitive development among primates, including humans. Combinatory object manipulation is a good indicator of cognitive development; it starts approximately when a human is 10 months old (Tanaka and Tanaka 1982). Three mother-reared chimpanzees in a captive setting initiated it at 8–11 months of age, which was comparable to that in humans (Hayashi and Matsuzawa 2003). Among its four types that were performed in the previous study, inserting actions were commonly observed in chimpanzees from an early age, while stacking-block behaviors started later, as compared to humans.

The three mother-reared chimpanzees were constantly exposed to their mothers’ stacking-block behaviors during the initial 3 years of their lives without any feedback from humans (Hayashi and Matsuzawa 2003; Hayashi 2007a). Among them, a female chimpanzee named Pal started stacking up blocks when she was 2 years and 7 months old. All three chimpanzees began performing this action when human experimenters conducted active training with positive reinforcements; this began when they were 3 years and 1 month old (Hayashi 2007a). After the stacking action’s acquisition, both chimpanzees and humans demonstrated similar performances in tasks using blocks of various shapes that were designed to assess their understanding of physical causality regarding combinatory manipulation (Hayashi and Takeshita 2009a).

## Nesting-cup tasks and human language

Furthermore, the nesting-cup tasks require insertion actions, a form of combinatory object manipulation. Greenfield et al. (1972) first applied it to assess cognitive development in human children aged 0–3 years. It was found that the same developmental order existed in both manipulative strategies and certain grammatical constructions. Human infants begin to combine two cups together (pairing), develop to combine three or more cups by sequentially adding a single cup to an existing unit of cups (pot), and then, combine three or more cups by manipulating an existing unit of multiple cups to be combined with a cup or unit (subassembly) (see Fig. 2 for an illustration). The authors specifically focused on the “subassembly” strategy, wherein a previously constructed structure consisting of two or more cups is moved as a unit into or onto another cup or cup structure. The subassembly strategy appeared in the final stage of development (observed only after 20 months in human children); thus, it is the most advanced combinatory strategy (Greenfield 1972). Greenfield (1991) elaborated on this idea and suggested that a common neural substrate underlies the hierarchical structure in the development of human language and combinatory object manipulation, including tool use. The definition of the subassembly strategy is similar to that of merge in human linguistics. Application of the subassembly strategy largely increases the flexibility of the sequential order of cup combinations. If one is only employing the pot strategy to successfully seriate all provided cups into a nesting structure with the least number of moves, one needs to judge the relative size of the cups and start inserting the second largest cup into the largest cup and so on, without an error. However, if one employs the subassembly strategy, one can start by pairing any two similar-sized cups to make a unit as the first step and continue to judge the similarity of sizes between the two items.

The nesting-cup tasks have been applied to nonhuman primates as a comparative scale of cognitive development (Matsuzawa 1991; Johnson-Pynn et al. 1999; Johnson-Pynn and Fragaszy 2001; Fragaszy et al. 2002). However, the subassembly strategy was employed by all the tested species of cebus monkeys and great apes that possess the ability of combinatory object manipulation (although the frequency of subassembly strategy was not clear in some studies) and failed to illuminate the species differences. These results may be explained by the fact that the original categories used by Greenfield (1972) were applied by focusing on the presence of the subassembly strategy during the entire process of making the final structure. Thus, to assess the process of crafting hierarchical combinations, a new notation system of action grammar was invented to reveal additionally precise patterns in their sequential combination (Hayashi 2007b). Three elements of object manipulation were extracted: which “object” was manipulated in which “action” and related to which “location.” In contrast with previous studies that judged the entire process for categorization, each combinatory object manipulation was categorized into either pairing, pot, or subassembly strategies (see Methods for an example). Accordingly, the efficiency of cup combinations was assessed and compared in humans and chimpanzees of various ages (Hayashi and Takeshita 2009b); both indicated similar trial-and-error strategies in creating hierarchical combinations among multiple nesting cups.

## Hierarchical complexity in object manipulation

Regarding hierarchical complexity, previous studies have reported varying results in different circumstances. Matsuzawa (1996) applied the “tree structure analysis” to illuminate the object manipulation’s hierarchical structure and tool use among wild chimpanzees. The latter’s most frequent patterns were categorized as “Level 1,” where an object (tool) is merged/combined with another one (target) with a single combinatory relation. A group of wild chimpanzees in Bossou, Guinea, West Africa, used a pair of stones to crack open nuts; this falls into the “Level 2” category, where a nut (target) is placed on an anvil stone (the first tool) and subsequently hit by a hammer one (the second tool) using two kinds of combinations among three objects. Additionally, infrequent occurrences of “Level 3” tool utilization were reported in the Bossou chimpanzees, where a wedge stone (the first tool) supported an anvil one (the second tool); a nut (target) was positioned on the latter and smashed using a hammerstone (the third tool) with three combinations of four objects. Although the intentional insertion of the wedge stone has been questioned in chimpanzees (Hayashi 2015), this was considered as having the most hierarchically complex structure in the tool-using behavior reported in wild chimpanzees.

Contrastingly, the nesting-cup tasks applied to captive chimpanzees indicated that a considerably higher level of hierarchical complexity could be achieved in terms of the number of items combined into a nesting structure (Hayashi 2007b). The task provides clear feedback on success/failure regarding cup seriation. Moreover, when multiple cups are combined into a nested structure, it can be moved as a unit to be merged with other cup(s), realizing the “subassembly” strategies. These two factors may have promoted more cups to be added into the nesting structure, enabling the maximum record of conjoining up to ten cups in a seriation performed by a female chimpanzee named Ai, who received artificial language training (Matsuzawa 1991). If we focus only on the number of combined objects, a clear difference is observed between the achievements in the wild and captive task settings. In this study, we attempted to highlight the hierarchical structure and its complexity in object manipulation and tool use during cognitive development in human and nonhuman primates.

## Scopes of the two studies

The great apes are the most closely related evolutionary relatives of human beings and are categorized in the same “Hominidae” family. However, the data on their cognitive development observed in object manipulation by the great apes other than chimpanzees have been limited (Bard 1995; Gomez 1999). Thus, the first part of this study aimed at comparing the developmental trajectory of combinatory object manipulation in human children and great ape infants under captive settings by using identical tasks to gain data on broader species-based comparison. In the current study, we conducted a longitudinal investigation of humans and chimpanzees as well as a cross-sectional examination of three other great apes (two bonobos, three gorillas, and four orangutans) in captive settings by using object-manipulation tasks that required either inserting or stacking combinatory actions. These two types of combinatory object manipulations appeared at different times during the development of chimpanzees (Hayashi and Matsuzawa 2003). We discussed the developmental data gained in the captive task settings regarding the tool-use literature on the wild great apes.

This study’s second part assessed the hierarchical complexity exhibited in the nesting-cup task to examine the highest cognitive capacities achieved by human children and adult chimpanzees. We examined the hierarchical structure building capacities (see Asano 2021, in this volume, for a review of this topic in the evolution of language and music) by applying the notation system of action grammar that focuses on the combinatory strategies used in sequential cup manipulations (Hayashi 2007b). We aimed to contribute to language evolution research by investigating the cognitive capacities required to build highly hierarchical combinations using multiple objects in human children and adult chimpanzees.

## [Study 1] Methods

### Subjects

The first study focused on the developmental trajectory of human children and great apes in comparative task settings providing identical objects. The subjects of the longitudinal study were three infant chimpanzees (*Pan troglodytes*). The chimpanzee infants (one male, Ayumu, and two females, Cleo and Pal) were raised by their biological mothers at the Primate Research Institute, Kyoto University, and examined once in 1–2 weeks from age 0 to 4 years.

**Fig. 1:**
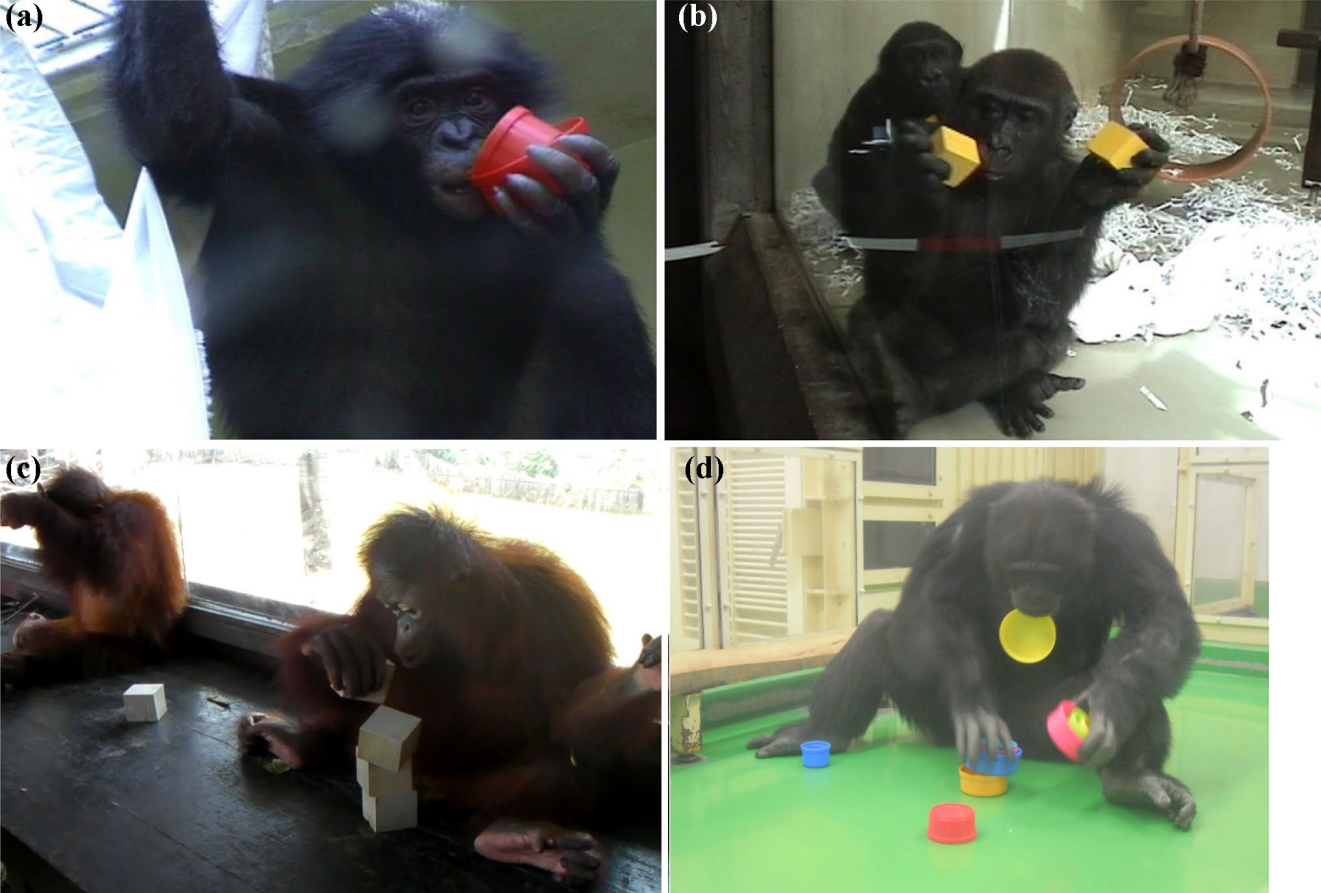
**a** A bonobo (Limbuko) manipulating a cup-unit. **b** A gorilla (Iringa) manipulating a block in each hand. **c** An orangutan (Deepa) stacking blocks. **d** A chimpanzee (Ayumu) participating in the nesting-cup task

The subjects of the cross-sectional study were two bonobo infants (*Pan paniscus*), three gorilla infants (*Gorilla gorilla*), and four orangutan infants (*Pongo pygmaeus pygmaeus*) (Figs. 1a, b, c, respectively). At the time of testing in August 2000, the bonobos and gorillas were housed at the Wilhelma Zoo in Stuttgart, Germany, in the nursery peer group for each species. Furthermore, during the August 2010 testing, the orangutans were accommodated at the Bukit Merah Orang Utan Island, Perak, Malaysia, in a nursery peer group. They were hand-reared by human caretakers mainly for health reasons in early infancy and were practically the last generation to be separated from their conspecific mothers. Table 1 shows detailed information on the great ape subjects.

**Table 1:** Information of the great-ape subjects

Species	Name	Date of birth	Sex	Age at test
Chimpanzee	Ayumu	04/24/2000	Male	0–4 y, 20 y
	Cleo	06/19/2000	Female	0–4 y, 20 y
	Pal	08/09/2000	Female	0–4 y, 20 y
Bonobo	Limbuko	10/04/1995	Male	4 y, 9 m
	Kuno	11/26/1996	Male	3 y, 8 m
Gorilla	Luena	12/25/1996	Female	3 y, 7 m
	Kumbuka	11/15/1997	Male	2 y, 8 m
	Iringa	01/31/1998	Female	2 y, 6 m
Orangutan	Deepa	11/08/2007	Female	2 y, 9 m
	April	04/13/2008	Female	2 y, 4 m
	Tuah	03/26/2009	Male	1 y, 5 m
	June Jr	12/01/2008	Female	1 y, 8 m

*y* year, *m* month

### Settings

The object-manipulation activities were provided to the subjects. Three tasks were selected from nonverbal tasks included in the Kyoto Scale of Psychological Development (KSPD) reported in Ikuzawa (2000): (1) stick insertion, (2) nesting cups, and (3) block stacking. As the KSPD tests have been standardized by testing 1562 Japanese children (ranging from 1 to 13 years of age), we used the data provided in KSPD showing the age of 50 and 75% of human children pass each task. The chimpanzee infants participated in similar tasks longitudinally under a face-to-face paradigm. Other great apes partook in the task comprising (2) nesting cups and (3) block stacking in a group setting. (1) The stick insertion involved inserting a rod into a hole on a box surface (reported as the “Box” task in Hayashi and Matsuzawa 2003). (2) The nesting cups were employed to insert cup(s) with different diameters in a seriated structure. (3) The block stacking was utilized to stack cubic blocks (5 × 5 × 5 cm, while KSPD tests used cubic blocks of 2.5 × 2.5 × 2.5 cm) into a vertical structure. Sometimes, we mixed cylindrical blocks (5 cm in height and 5 cm in diameter) in the provided set of blocks to test their understanding of physical causality (Hayashi 2007a).

The chimpanzee infants were accompanied by the mother and tested in a face-to-face setting by a human experimenter at a playroom at the Primate Research Institute, Kyoto University. Chimpanzee infants were assessed longitudinally, and their mothers performed object manipulation tasks as models for the infants. Chimpanzees sporadically received food rewards during the face-to-face tasks regardless of their performance in object manipulation.

The other great apes were tested in a group setting with their living enclosure with peers during a period of 2–3 weeks. The subjects participated only in tasks (2) and (3) because of limitations regarding time and resources. A keeper or the first author sporadically entered the enclosures to provide the objects to be manipulated or to model manipulative behaviors. No food rewards were awarded during the observations in the group setting.

### Analysis

The human data used for the analysis of species comparison in Study 1 were mainly based on the larger data set from the KSPD reported in Ikuzawa (2000). The data set for chimpanzee subjects were based on the longitudinal observations. Moreover, the species comparison in Study 1 employed cross-sectional data from three species of the great apes by focusing on the combinatory manipulation onset (more general manipulative repertoire of bonobos and gorillas was reported by Hayashi and Takeshita (2002) and Hayashi et al. (2006)).

## Results

Table 2 summarizes the developmental onset of each behavioral milestone in each species. The human data in it are based on the data from the KSPD reported by Ikuzawa (2000), except the data on making higher tower with larger cubic blocks and stacking cylindrical blocks which was gained from the subjects of the Study 2. Half of the human children succeeded in the stick insertion at 1 year and 1 month of age. A chimpanzee, Pal, initially succeeded it at 8 months of age. Similarly, half of the human children prospered in the insertion to make a three-cup nesting at 1 year and 5 months of age; further, a chimpanzee, Cleo, first succeeded at 1 year and 5 months of age. Thus, the human children and the chimpanzees demonstrated a combinatory object manipulation requiring an inserting action from a similar chronological age. Contrastingly, half of the former thrived in stacking smaller cubic blocks at 1 year and 1 month of age, while a chimpanzee, Pal, did so in stacking larger cubic blocks at 2 years and 7 months of age. Although the onset of stacking actions was delayed, the chimpanzee demonstrated rapid improvements in the first month after the initial success in stacking blocks and started to do well in making a higher tower of seven blocks. Contrarily, the human children succeeded in making a tower of seven blocks of larger size only after about 8 months from their first success (at 1 year and 9 months of age) with the same cubic blocks (5 × 5 × 5 cm). Additionally, two other infant chimpanzees primarily triumphed in stacking larger cubic blocks at 3 years and 1 month of age when the training attempts were introduced by the human experimenters. After the success in stacking cubic blocks, both human children and the chimpanzee who spontaneously piled them succeeded in assembling cylindrical blocks from initial phase (reported precisely by Hayashi 2007a).

**Table 2:** Age at the first success in each type of combinatory manipulation

	Success in stick insertion	Success in cup insertion	Success in block stacking	Success in 7-block stacking	Success in cylinder stacking
Human 50%	1 y, 1 m	1 y, 5 m*	1 y, 1 m	1 y, 9 m	1 y, 3 m
Chimpanzee	0 y, 8 m	1 y, 5 m*	2 y, 7 m	2 y, 8 m	3 y, 2 m
Bonobo	–	3 y, 8 m	4 y, 9 m	4 y, 9 m	N
Gorilla	–	3 y, 7 m	2 y, 6 m	(3 y, 7 m)	3 y, 7 m
Orangutan	–	2 y, 9 m	2 y, 9 m	N	N

Single underlined: Data retrieved from KSPD, Ikuzawa (2000) Double underlined: Data gained from the same human participants as Study 2. For human children, we showed the age of more than 50% of subjects succeeded in the combinatory manipulation

*y* year, *m* month

*Age at success in combining three cups

() Age at success in stacking five blocks

Moreover, the other three species of the great ape infants showed both types of combinatory object manipulation, insertion, and stacking in captive settings during their development. However, the timing and the order of development in the different types of combinatory object manipulations varied among the great apes. The bonobos displayed patterns of acquisition order similar to those of chimpanzees: a younger bonobo subject, Kuno, aged 3 years and 8 months, successfully inserted a cup into a larger cup but did not stack up the blocks. The older bonobo subject, Limbuko, aged 4 years and 9 months, successfully combined up to three cups into a nesting structure and succeeded in creating a tower of seven blocks. In contrast, a gorilla aged 3 years and 7 months old, named Iringa, began block stacking but not cup insertion. Only the oldest gorilla subject, Luena, aged 3 years and 7 months, succeeded in stacking up five blocks and combining up to three cups in a nesting structure. An orangutan aged 2 years and 9 months, Deepa, succeeded in both cup insertion and block stacking.

## [Study 2] Methods

### Subjects

For the second study of the nesting-cup manipulation, the participants of the longitudinal study were 18 human children (eight males and ten females, aged 1 year and 0 months to 4 years and 0 months). They were recruited from the Umikaze Infant Laboratory of the University of Shiga Prefecture and tested once in 1- to 3-month intervals. We attempted to include two participants (one male and one female) from each age class in months above 1 year; however, this was occasionally difficult with the younger age classes. For the chimpanzee subjects, we evaluated the same chimpanzees after 20 years from the onset of Study 1. Additionally, we tested other adult chimpanzees (Ai, Chloe, and Pan, the mothers of the three infant chimpanzees).

### Settings

The human children were accompanied by a parent and examined in a face-to-face setting with the first author under the consent of their parents and approval from the University of Shiga Prefecture. The three chimpanzees were continuously tested in the nesting-cup task in a face-to-face paradigm with the first author, once a month, after the period covered by Study 1 until they were 9 years old. The average number of cups successfully combined into a nesting structure was 4.97, 4.69, and 5.97 for Ayumu, Cleo, and Pal, respectively, when they were 6.5–10 years old. For the tests in Study 2, we arranged cups in an experimental booth and allowed a chimpanzee to access and manipulate the objects (Fig. 1d). Both human and chimpanzee subjects were given verbal encouragement for the general manipulation of objects; however, they were not provided any instructions for specific manipulative strategies. The chimpanzee participants in the nesting-cup task received a food reward after each trial regardless of their performances in completing the full seriation.

### Analysis

**Fig. 2:**
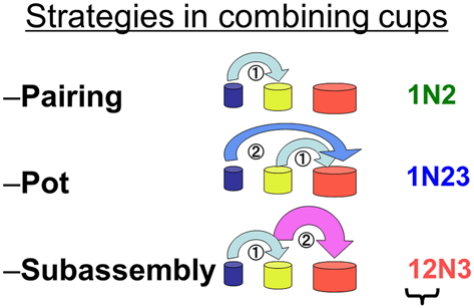
Schematic illustration of three types of combination strategies. The example codes in the right side of the figure show the code for the last manipulative action in each sequence

The analysis of the combination strategies in the nesting-cup task utilized the data gained from the face-to-face and individual tasks with the human children and the adult chimpanzees, respectively. A flow of cup manipulation was coded following the notation system of action grammar developed by Hayashi (2007b) that describes three key components of a manipulative action: object, action, and location. For instance, 1N2/12N3 is coded when a subject inserts the smallest cup into a second cup and subsequently adds the two cup-unit into the largest/third cup. The resulting coding data were further analyzed using the normal categorization of combination strategies defined by Greenfield et al. (1972) for each combinatory action (see Fig. 2 for an illustration). Thus, the aforementioned instance was coded as “Pairing” (1N2) and “Subassembly” (12N3). Moreover, we added some additional categories to better describe the entire manipulative pattern, including piling or disassembly.

The human children above 1 year 0 months were divided into the following six age classes: 1 year 0 month–1 year 5 months (seven participants), 1 year 6 months–1 year 11 months (ten participants), 2 years 0 months–2 years 5 months (12 participants), 2 years 6 months–2 years 11 months (12 participants), 3 years 0 months–3 years 5 months (12 participants), and 3 years 6 months–4 years 0 months (14 participants). We selected the last trial in each participant that provided the largest number of cups to be seriated by them. The coded data in the last trial were categorized into nine manipulative patterns. Greenfield et al. (1972) defined three patterns of cup combinations: pairing, pot, and subassembly strategies. Additionally, we added further clarifications to the three classical strategies. We differentiated the target of an inserting action: “Subassembly” denotes inserting a cup-unit into a larger cup, while “Unit–Unit” indicates inserting a cup-unit into a larger cup-unit, categorized as the more advanced form of the subassembly strategy. We also differentiated piling, in which a larger cup was stacked on a smaller one. Since the piling combination was relatively rare, we made only two categories: “Pile,” piling a cup on a smaller cup/unit, and “Pile Sub,” a cup-unit on a smaller cup/unit. “Put” signifies the participant combined a cup/unit with another cup/unit, however, retrieved it without releasing the manipulating hand. “Disassemble” means the participant detached a cup/unit from a premade cup-unit. “Other” included any other manipulation of cup/unit such as mouthing, replacing on the floor surface, reversing the orientation of a cup, banging, etc. We calculated the percentage of each manipulative category divided by the total number of manipulative actions recorded in the last trial for each participant. We selected ten trials from the adult chimpanzee subjects, where they were given nine or ten cups to be seriated. Because of the limitation regarding the number of participants in each age class, we did not conduct any statistical analyses.

## Results

Table 3 shows the summary of the cup manipulations performed by human children and adult chimpanzees. The last trial with the largest number of cups provided in a testing session was analyzed for each human participant of a tested age. Human children were categorized into six age classes. The maximum number of cups provided in the last trial ranged from three to six in the age class starting from 1 year 0 months and reached nine or ten in the age class starting from 3 years 6 months. The maximum number of cups in a successful nesting structure (a smaller cup, regardless of its contiguity, is inserted in a larger cup) ranged from two to five in the age class starting from 1 year 0 months and reached nine or ten in the age class starting from 3 years 6 months. The success rate (percentage of trials in which all cups provided were successfully combined in a complete nesting structure) was 14.3% in the age class starting from 1 year 0 months and reached 100% in the age class starting from 3 years 6 months. Based on the notation system of action grammar defined by Hayashi (2007b), we recorded the manipulation patterns for the human children and the adult chimpanzees. The total number of manipulations counted in each trial ranged from 4 (observed in a subject of 1 year and 6 months old for the four given cups) to 140 (observed in a subject of 2 years and 9 months old for the nine given cups) for the human children. The average number of manipulations in a trial gradually increased, peaked at the age class starting from 2 years 6 months (about 61 manipulations were recorded in a trial), and gradually decreased in the older children. For the adult chimpanzees, the maximum number of cups was 9–10 and those successfully combined into a nesting structure was 5–10, with a success rate was 70.0%. The total number of manipulations totaled in each trial ranged from 23 to 192 (observed in Pal and Cleo for the nine given cups, respectively); the average was 102.8 for chimpanzees.

**Table 3:** Cup manipulations observed in human children and chimpanzees

Species	Age class	Max # of cups	Max # in a nesting	Success rates (%)	Min # manipulation	Max # manipulation	Average # manipulation	SD
Humans	1 y 0 m–1 y 5 m	3–6	2–5	14.3	5	37	14.9	10.7
	1 y 6 m–1 y 11 m	4–9	3–9	50.0	4	44	29.7	12.7
	2 y 0 m–2 y 5 m	5–9	4–9	41.7	19	81	37.6	16.8
	2 y 6 m–2 y 11 m	9–10	5–10	58.3	23	140	60.9	34.4
	3 y 0 m–3 y 5 m	7–10	5–10	83.3	16	67	37.2	17.0
	3 y 6 m–4y 0 m	9–10	9–10	100.0	10	60	27.6	13.8
Chimpanzees		9–10	5–10	70.0	23	192	102.8	59.2

Human children were categorized into six age classes. The columns show “Maximum number of provided cups,” “Maximum number of cups in a successful nesting structure,” “Success rates,” “Minimum number of manipulations performed in a trial,” “Maximum number of manipulations performed in a trial,” “Average number of manipulations in a trial,” and “Standard deviation of the number of manipulations in a trial”

**Fig. 3:**
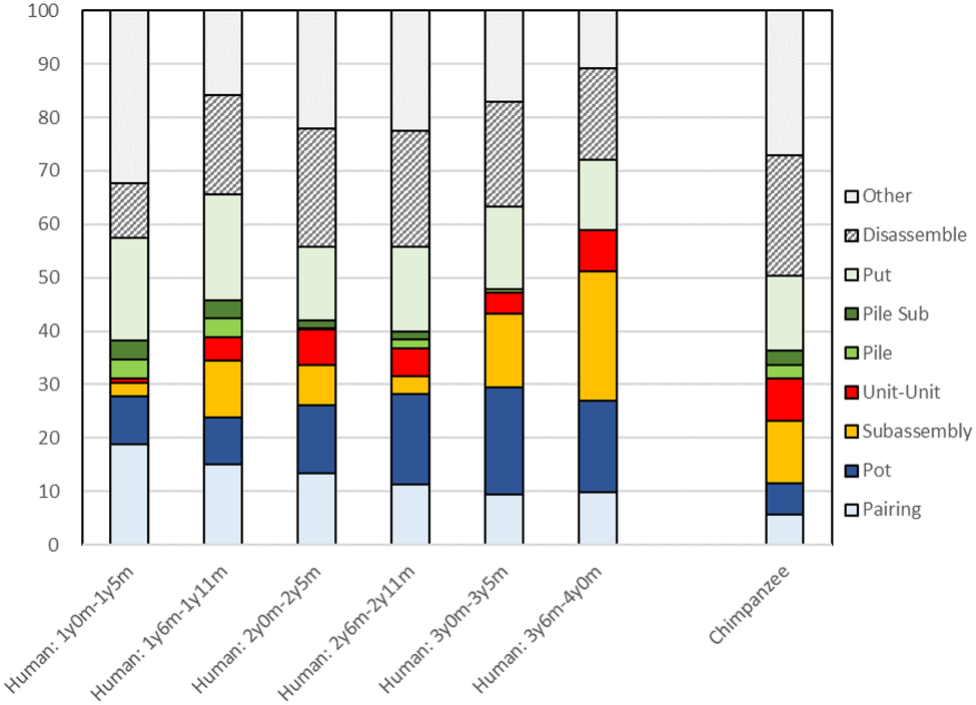
Proportion of the manipulative actions during nesting-cup task in human children of six age classes and adult chimpanzees

Figure 3 shows the percentage of each manipulation pattern for the human children of six age classes and the adult chimpanzees. In the former, the most popular combinatorial pattern was pairing (18.8%) for those aged 1 year and 0 months to 1 year and 5 months of age. The pot strategy gradually increased and peaked (20.1%) in human children from 3 years and 0 months to 3 years and 5 months of age. In the oldest age class of the human children from 3 years and 6 months to 4 years and 0 months, the classical subassembly strategy was observed in 31.9% (24.1% for the single recipient cup and 7.8% for the unit–unit combination). In adult chimpanzees, it was observed in 19.7% (11.6% for the single recipient cups and 8.1% for the unit–unit combinations). However, there were great individual variations in the use of the subassembly strategy regarding the chimpanzee subjects: Cleo showed none of the 192 manipulations, Pal displayed 11 subassembly strategies (nine for the single recipient cup and two for unit–unit combination) out of the 23 manipulations scoring the highest (47.8%) for the subassembly strategy (details are shown in Supplementary Table).

## General discussion

### Development of combinatory object manipulation in great apes

Combinatory object manipulation is a precursor of tool-using behaviors and a good indicator of cognitive development in primates, including humans. In Study 1, we conducted a cross-species comparison to accumulate additional data on the developmental pathway of combinatory object manipulation in the great apes. The tool-using behaviors in the wild great apes other than the chimpanzees are relatively infrequent compared to the wild chimpanzees; however, all great ape species examined in this study under captive settings readily showed two forms of combinatory object manipulation, insertion and stacking, during their infancy. Thus, they may share fundamental physical and cognitive abilities to enable the emergence of tool use in their wild habitats. Furthermore, a comparison among the great apes revealed that the insertion action in the chimpanzees develops at a time comparable to that of humans. The early acquisition of inserting action in the chimpanzees may also explain the tool-use commonality reported in the wild chimpanzees.

The action patterns involved in combinatory object manipulations may explain the differences observed between the results of humans and great apes. Human children started to exhibit two kinds of combinatory object manipulation, insertion, and stacking, at around the same age when they passed 1 year of age. Although chimpanzees started to demonstrate an inserting action from a comparable age with human children, the first acquisition of stacking action was delayed in the chimpanzees, as compared to the humans. A similar order of development was observed for the bonobos. In contrast, the gorillas started to display stacking actions at an earlier age. The orangutans exhibited both types of combinatory object manipulations simultaneously. Torigoe’s study (1985) reported action patterns used in object manipulation among 74 species of non-human primates by providing a rope and a wooden cube to be manipulated freely in their cages. However, it mainly focused on more general action repertoire and excluded the precise analysis on combinatory object manipulation among the great apes. Moreover, the described patterns of combinatory object manipulation were mainly targeted to an environmental substrate (such as a wire mesh of the cage or water surface) and not to a detached object, which was the present study’s focus. We need additional developmental data from a greater number of the great ape subjects under the setting of providing them with more detached objects to provoke combinatory manipulations.

Another important point for the observed order of the action patterns involved in combinatory object manipulation was the early acquisition of the inserting action in the manipulative development of captive chimpanzees. Among the long list of tool-use repertoire of the wild chimpanzees reported, the tool-using behaviors that include the inserting action are commonly observed in almost all chimpanzee communities across Africa (Whiten et al. 1999), except for a community in Budongo, Uganda. Despite efforts to find and solicit the use of stick tools even under experimental settings, the Budongo chimpanzees have never been reported to employ stick tools (Gruber et al. 2011; Mugisha et al. 2016) that have been commonly reported in other chimpanzee communities (Whiten et al. 2001). There might be some ecological or social/cultural factors that explain the absence of stick tool use in Budongo (Gruber et al. 2012; Lamon et al. 2018); nevertheless, even in the Budongo community, the chimpanzees use leaf tools with an inserting action (Gruber et al. 2016). Thus, this inserting action may be universal in the behavioral repertoire of chimpanzees supported by the ecological validity to survive in the wild with the assistance of inserting tools. This may explain the early acquisition of the insertion action even in captivity. However, the great ape species compared in this study revealed that the acquisition of inserting action in chimpanzees was early and the timing was comparable to that of human infants instead of other great apes.

### Patterns of object combinations and implications for tool-using behaviors in the wild great apes

Although the great apes possess the fundamental cognitive ability to perform combinatory object manipulation in captivity, some other factors may contribute to the tool-use frequency and variety in the wild. Furuichi et al. (2015) compared the tool-use repertoire in wild chimpanzees and bonobos and showed that the latter rarely use tools for feeding, while the former do. They also pointed out that the paucity of tool use in wild bonobos was not explained by the current ecological or social factors; however, they suggested the possible influence of past historical circumstances. Koops et al. (2015) reported that wild chimpanzees engaged more in object manipulation and play than bonobos of the same age range of zero to eight years, consistent with a species difference in the intrinsic motivation for tool use. However, in captive settings, both chimpanzees and bonobos have shown equally diverse and highly complex tool-use repertoires in most contexts (Gruber et al. 2010). Similarly, the tool-use repertoires in gorillas (Breuer et al. 2005) and orangutans (Meulman and van Schaik 2013; van Schaik et al. 2003) in the wild have been relatively smaller than those in chimpanzees (Sanz and Morgan 2007). Nevertheless, the rehabilitated and captive orangutans have demonstrated various tool-using behaviors, including complex imitative actions (Russon and Galdikas 1993; Damerius et al. 2019). Some previous studies have proposed hypotheses to explain the species differences found regarding tool-using behavior (Byrne 2004; van Schaik et al. 1999). Our study showed that the captive great apes readily acquire combinatory object manipulation during their infancy; moreover, they may share the common cognitive abilities that enable the emergence of tool-using behaviors under certain conditions in wild settings.

It should be noted that there were species differences in the order of development between the two types of combinatory object manipulation. Previous developmental studies have illuminated it with respect to performing nut cracking behavior. The chimpanzees in Bossou started to put a nut on an anvil stone and hit something with a hammerstone at the same age of one and a half years (Inoue-Nakamura and Matsuzawa 1997). Contrastingly, the tufted capuchin monkeys in the Tietê Ecological Park, Brazil, began smashing an object against a substrate (first level of combinatory object manipulation) from as early as age five months; however, placing a nut on a surface appeared considerably later, at Age Block 4, ranging from one and a half to two years of age (Resende et al. 2008). Thus, even if the final behavioral outcome is identical, ontogeny may vary among different species. Although we had limited number of subjects at certain age points, our study suggested that each great ape species may exhibit specific patterns in the developmental order of different types of combinatory object manipulations. Specifically, gorillas showed the opposite order of development from chimpanzees and bonobos, an earlier appearance of stacking blocks as compared to inserting cups. This may reflect the species-specific patterns of manipulative tendency, as wild gorillas are known to have relatively limited tool-using repertoires; however, they indicate highly sophisticated bimanual coordination and digit role differentiation in processing foods (Byrne et al. 2001). Another possibility is that the objects used in the present study may have provided diverse affordances for different species of the great apes. Human infants develop tool-using behaviors through exploratory manipulation by detecting and relating affordances between objects (Lockman 2000). For chimpanzees and bonobos, the affordances of cups might be more salient and trigger manipulative actions, including inserting action to make a unit of multiple cups. The younger bonobo subjects demonstrated additionally varied manipulation of cups (Hayashi and Takeshita 2002) and prepared cup combinations and transported them as units to other locations. For gorillas, the affordances of blocks may be more prominent and activate various manipulative actions, including stacking blocks into a tower structure. We should accumulate more empirical data through longitudinal studies focusing on comparative cognitive development among primates (Biro et al. 2006; Meulman et al. 2013; Rosati et al. 2014) both in captivity and in the wild. However, we should avoid testing infants of great apes who were separated from their conspecific mothers and peers to foster the development of species-specific behaviors (Hayashi and Matsuzawa 2017). Similar to the situation in the wild, we were able to observe the development of infants’ manipulative skills in group settings including the mothers by considering the effect of social learning.

### Hierarchical combinations of nesting cups in adult chimpanzees and human children

In Study 2, we demonstrated the clear advantage of explicit experimental settings with positive reinforcement for assessing the maximum performance in making hierarchical object combinations. The process of crafting highly hierarchical combinations can be regarded as “action grammar,” which may be linked to the cognitive underpinnings of human linguistic abilities. The wild chimpanzees in Bossou, Guinea, select and reuse effective tool composites and certain functional units of stone tools (Carvalho et al. 2009). The maximum number of objects assembled in a tool composite was four that was observed during nut cracking by the Bossou chimpanzees; two wedge stones under an anvil, and a hammerstone (Carvalho et al. 2008). Contrastingly, the captive adult chimpanzees succeeded in combining up to 10 cups into a nesting structure. The clear sensorimotor feedback of the success/failure of an inserting action may facilitate the creation of various combinations of cups with trial-and-error strategies. Moreover, the combined cups can be easily handled as a unit, which is in clear contrast to stone or block composites. Providing these objects designed to solicit more complex and hierarchical combinations as a set in an individual experimental setting may lead the chimpanzees to concentrate on cup manipulation and elaborate the cup combinations. Both chimpanzees and human children were offered social encouragement to manipulate the cups as well as social praise at the end of each trial (moreover, a food reward for chimpanzee subjects). Although the social encouragement did not seem to enhance the specific manipulative strategy, it may prolong the time of cup manipulations in general, providing more chances for achieving the final goal through repetitive trial-and-error strategies. Collectively, the nesting-cup tasks increased the probability of achieving an additionally complex hierarchical structure by a sequential combinatory manipulation.

The evolutionary comparison of complexity in animal and early human tool use showed that the highly hierarchical tool use and the manufacture enabled by the working-memory capacity characterizes the intelligence observed only in early humans (Haidle 2010). The increased complexity of stone tool-making technology observed in the human lineage has also been assessed (Stout 2011) and further discussed in line with the technological pedagogy hypothesis, which led to the emergence of intentional vocal communication among humans (Stout and Chaminade 2012). However, more focus should be given to the sequence of actions employed to make complex tools that can be analyzed as action grammar (Pastra and Aloimonos 2012; Stout et al. 2018). Hansell and Ruxton (2008) claimed that tool utilization (originally defined as using a detached object) should be examined within the wider context of construction behaviors (such as nest building) observed in a wider range of animals. Construction behaviors usually require multiple steps during the building phase, however, they are all categorized into the “pot strategy,” sequentially adding the subsequent element without a role reversal. The role reversal or recursion in linguistics can only be realized by manipulating multiple detached objects as a unit in the sequence of combinatory object manipulation in the form of a “subassembly strategy” (Fujita 2009, 2017). Five out of six adult chimpanzees tested in the current nesting-cup task readily employed the subassembly strategy that may require some level of cognitive capacity to manipulate a unit of multiple objects. Older human children above three years old also employed the subassembly strategy, and the success rates were increased in the same age classes. Moreover, those human children and adult chimpanzees, who demonstrated the subassembly strategy, also combined a unit of cups with another unit (unit–unit combination) that may be more cognitively demanding to divide attention into two equal parts (referred to as “multiple attention” in Fujita and Fujita’s study (2021). However, it should be noted that not only the human children aged above 3 years but also those aged below it showed a subassembly strategy during the manipulative sequence. The adult chimpanzees who participated in this study had prior experiences in various types of cognitive experiments, including object-manipulation tasks. Not only a chimpanzee named Ai, who had received artificial-language training, but also other chimpanzees reported subassembly strategies (referred to as “Action-Merge” by Fujita and Fujita (2021)) if they were requested to combine cups to construct a hierarchical structure.

Study 2 of the present study showed that adult chimpanzees experiencing a complex problem-solving task of nesting cups readily made highly complex hierarchical combinations among cups and moved the combined cups as a unit to be further merged with another one. The object manipulation may have worked as an external enhancer to achieve a hierarchical complexity in cognition and behavior in both human children and adult chimpanzees. Both species showed similar patterns of adjusting the cup combinations through trial and error; however, they also used the subassembly strategy that was suggested to be linked to a human language (Greenfield 1991). The manipulation of objects can be regarded as the externalization of internal/cognitive processing; its result becomes visible as the change in the environment. Thus, compared to the completely internal processing of the human linguistic structure, the hierarchical structure-building during object manipulation may be more advanced and widely confirmed from the early stages of development and evolution of human beings. We need to further investigate the sequential order of each manipulative action exhibited in the nesting-cup task to emphasize species-specific and age-related changes in the patterns of action grammar. Our study suggests that chimpanzees may share cognitive underpinnings for creating hierarchical combinations among multiple objects through sequential manipulative actions. We need to clarify which cognitive functions play a key role in accelerating the evolution of human language by applying comparable tasks in different species of Hominidae.

## Conclusions

We found that great-ape infants readily develop cognitive capacities that enable the emergence of combinatory object manipulations, which may be regarded as action merge. Great apes, especially chimpanzees, utilize tools in their wild habitat based on this fundamental ability of combining objects. Study 1 showed that the developmental order of the two types of combinatory object manipulation, insertion and stacking, were different among the great apes; however, we need more longitudinal developmental studies to ascertain the precise timing of acquisition in species other than chimpanzees.

In Study 2, we found that both human children and adult chimpanzees could combine nesting-cups to make a highly hierarchical structure. However, according to the individual action-based analysis grounded upon the notation system of action grammar developed by Hayashi (2007b), human children and adult chimpanzees demonstrated not only combinatory object manipulations but also disassembling manipulations on the previously made combinations of cups, indicating the use of the trial-and-error strategy. By applying the subassembly strategy, human and chimpanzee subjects may try to improve the cup combinations more efficiently.

Taken together, object-manipulation tasks are useful for comparing human and nonhuman primates in comparable task-settings and may solicit highly complex combinatory manipulations as objects works as an external enhancer. Comparative studies on the development of combinatory object manipulation and material intelligence in primates may shed further light on the cognitive capabilities which enabled the evolution of human language.

## Supplementary Information

Below is the link to the electronic supplementary material.

Supplementary file1 (PDF 212 KB)
