# Supplementary material for: Hierarchical object combination and tool use in the great apes and human children
Source: Primates. 2022 Aug 1;63(5):429–41. doi: 10.1007/s10329-022-01003-2 (PMC9463204; doi:10.1007/s10329-022-01003-2)
Supplement: Supplementary file 1 — Supplementary file1 (PDF 212 KB) [file 10329_2022_1003_MOESM1_ESM.pdf]

Supplement: Manipulations observed in the nesting-cup task for human infants and adult chimpanzees

Human: ID	Age	Given #	Max #	Result	Total	# Pairing	# Pot	# Subassem	# Unit-Unit	# Pile	# Pile Sub	# Put	# Disassem	# Other
M01	1y0m	4	3	F	13	2	1	0	0	1	0	1	2	6
M01	1y1m	4	2	F	11	2	0	0	0	0	1	2	0	6
M02	1y2m	3	2	F	15	4	0	0	0	0	0	5	2	4
M01	1y2m	4	2	F	5	2	0	0	0	0	0	0	1	2
M02	1y3m	6	3	F	37	5	0	2	0	0	0	17	4	9
M01	1y3m	5	4	F	6	0	3	0	0	1	1	0	0	1
M02	1y5m	5	5	S	17	3	1	2	1	0	0	5	2	3
M02	1y6m	6	6	S	38	6	7	2	0	0	0	9	10	4
F01	1y6m	4	3	F	4	1	0	0	0	1	1	1	0	0
M02	1y7m	9	8	F	26	4	1	3	4	0	0	5	5	4
M03	1y7m	9	9	S	41	4	6	1	2	0	0	11	5	12
M02	1y9m	9	9	S	28	4	1	6	2	0	0	8	5	2
M03	1y9m	5	4	F	29	5	4	2	0	0	0	9	6	3
M02	1y10m	9	9	S	17	3	0	7	1	0	0	1	3	2
F02	1y10m	4	3	F	26	4	1	0	1	1	0	6	5	8
M02	1y11m	8	8	S	44	6	5	5	3	0	0	2	12	11
F02	1y11m	9	4	F	44	3	8	3	0	3	3	5	11	8
M02	2y0m	9	9	S	39	2	5	6	5	0	0	8	10	3
F02	2y0m	5	4	F	38	7	7	0	1	0	2	4	13	4
M02	2y1m	9	9	S	19	2	2	5	2	0	0	2	3	3
F03	2y1m	9	6	F	81	6	18	1	2	0	1	19	20	14
M03	2y2m	9	9	S	26	3	1	2	2	0	0	1		17
F03	2y2m	9	6	F	35	5	7	1	1	0	0	5	6	10
M03	2y3m	9	9	S	52	3	10	0	5	0	0	6	10	18
F04	2y3m	5	4	F	26	6	2	0	1	0	1	3	7	6
M04	2y4m	9	6	F	44	8	5	2	2	0	0	8	9	10
F04	2y4m	9	5	F	43	9	2	1	2	1	2	10	10	6
M04	2y5m	9	5	F	31	3	4	8	1	0	0	3	9	3
F04	2y5m	9	9	S	50	8	5	1	8	0	1	5	14	8
M04	2y6m	9	9	S	49	5	6	4	6	0	0	8	13	7
F03	2y6m	9	9	S	73	7	10	4	3	0	0	12	18	19
M04	2y7m	9	8	F	85	13	1	5	6	0	1	19	18	22
F04	2y7m	9	5	F	25	6	2	0	3	1	1	2	5	5
M05	2y8m	9	5	F	23	1	4	2	0	3	2	0	3	8
F05	2y8m	9	9	S	25	3	9	0	0	0	0	4	4	5
M04	2y9m	9	6	F	49	6	6	2	1	0	0	15	8	11
F06	2y9m	9	7	F	140	13	22	3	6	2	0	33	39	22
M06	2y10m	9	9	S	45	5	13	0	1	0	0	4	11	11
F03	2y10m	10	10	S	63	4	16	0	3	0	0	13	13	14
M04	2y11m	10	10	S	55	7	8	3	4	0	0	9	13	11
F07	2y11m	10	10	S	99	8	19	0	6	2	2	11	26	25
M07	3y0m	10	10	S	36	3	9	1	3	0	0	6	7	7
F05	3y0m	10	10	S	55	2	19	1	0	0	0	7	13	13
M06	3y1m	10	10	S	25	4	3	2	2	0	0	5	2	7
F04	3y1m	9	5	F	21	2	5	5	0	0	0	2	5	2
M04	3y2m	10	10	S	23	3	6	2	2	0	0	4	3	3
F06	3y2m	10	10	S	34	1	16	0	0	0	0	3	8	6
M07	3y3m	10	10	S	40	4	10	3	3	0	0	4	10	6
F07	3y3m	10	10	S	31	2	11	0	0	0	0	6	4	8
M05	3y4m	7	5	F	67	9	7	2	0	5	1	15	16	12
F08	3y4m	9	9	S	32	3	0	10	3	0	0	5	8	3
M08	3y5m	10	10	S	16	1	0	10	0	0	0	1	2	2
F08	3y5m	10	10	S	66	9	1	10	4	0	0	17	16	9
M07	3y6m	10	10	S	47	6	5	3	8	0	0	7	13	5
F05	3y6m	10	10	S	29	3	10	0	0	0	0	5	4	7
M06	3y7m	10	10	S	60	7	6	9	3	0	0	10	16	9
F09	3y7m	10	10	S	29	2	11	0	2	0	0	5	6	3
M06	3y8m	10	10	S	21	4	0	5	3	0	0	3	3	3
F09	3y8m	10	10	S	43	2	15	1	0	0	0	10	9	6
M08	3y9m	10	10	S	12	3	1	5	1	0	0	1	1	0
F10	3y9m	10	10	S	26	2	4	8	1	0	0	2	6	3
M08	3y10m	10	10	S	11	1	0	8	0	0	0	2	0	0
F10	3y10m	10	10	S	33	1	11	1	4	0	0	2	8	6
M08	3y11m	10	10	S	18	1	0	9	1	0	0	4	2	1
F10	3y11m	9	9	S	25	2	5	3	5	0	0	1	7	2
M08	4y0m	10	10	S	10	1	0	8	0	0	0	1	0	0
F05	4y0m	10	10	S	25	1	9	0	4	0	0	1	5	5

Chimp: ID	Age	Given #	Max #	Result	Total	# Pairing	# Pot	# Subassem	# Unit-Unit	# Pile	# Pile Sub	# Put	# Disassem	# Other
Ai(F)	44y	9	9	S	39	4	2	4	4	1	1	7	8	8
Ai(F)	44y	10	10	S	95	9	3	6	13	1	4	14	26	19
Chloe(F)	39y	9	9	S	99	6	4	9	10	2		31	21	16
Pan(F)	36y	9	9	S	79	4	3	10	6	2	3	10	19	22
Ayumu(M)	20y	9	9	S	112	6	14	2	3	5	5	12	24	41
Ayumu(M)	20y	10	5	F	188	8	16	4	4	6	15	25	41	69
Cleo(F)	20y	9	7	F	192	10	28	0	0	13	3	8	42	88
Pal(F)	20y	9	9	S	23	1	1	9	2	0	0	2	5	3
Pal(F)	20y	9	8	F	151	4	4	19	15	2	4	19	33	51
Pal(F)	20y	10	10	S	50	2	0	11	8	0	0	7	12	10
